# Association of diabetic peripheral arterial disease and objectively-measured physical activity: NHANES 2003-2004

**DOI:** 10.1186/2251-6581-13-63

**Published:** 2014-05-27

**Authors:** Paul D Loprinzi, Kalen Abbott

**Affiliations:** 1 Departments of Exercise Science and Physical Therapy, Donna & Allan Lansing School of Nursing & Health Sciences, Bellarmine University, Louisville, KY 40205, USA; 2 Maricopa Integrated Health System, Phoenix, AZ, USA

**Keywords:** Accelerometry, Epidemiology, Exercise, National Health and Nutrition Examination Survey (NHANES)

## Abstract

**Background:**

Although much is known about the management of peripheral arterial disease among adults in the general population, the management of this disease among those with diabetes, and the effects of diabetic-induced peripheral arterial disease on *objectively-measured* physical activity, is unclear. Here, we examined the association between accelerometer-assessed physical activity and peripheral arterial disease among a national sample of U.S. adults with diabetes.

**Methods:**

Data from the 2003–2004 National Health and Nutrition Examination Survey were used. Physical activity was measured using an accelerometer in 254 adults with diabetes. Peripheral arterial disease was assessed via ankle brachial index. Negative binomial regression analysis was used to examine the association between physical activity and peripheral arterial disease.

**Results:**

Results were adjusted for age, gender, race-ethnicity, comorbidity index, smoking, HgbA1C, C-reactive protein, homocysteine, glomerular filtration rate, microalbuminuria, peripheral neuropathy, physical functioning, and medication use. After adjustments, participants with peripheral arterial disease engaged in 23% less physical activity (RR = 0.77, 95% CI: 0.62-0.96) than those without peripheral arterial disease.

**Conclusions:**

These findings demonstrate an inverse association between accelerometer-assessed physical activity and peripheral arterial disease in a national sample of U.S adults with diabetes.

## Introduction

Peripheral arterial disease, often assessed as the ratio of systolic blood pressure in the upper and lower extremities, is a condition characterized by atherosclerotic occlusive disease of the lower extremities [1], and is considered a major risk factor for poor quality of life, lower-extremity amputation and cardiovascular morbidity and mortality [2]. Prevalence estimates of peripheral arterial disease are substantially higher for adults with diabetes (range: 9.5%-29%), compared to those without diabetes (4.5%) [3]. Indeed, it has been shown that diabetes, along with smoking, are main risk factors for developing peripheral arterial disease [4]. Although much is known about the management of peripheral arterial disease among adults in the general population, the management of this disease among those with diabetes is less clear [1]; for example, peripheral arterial disease is more subtle in its presentation in individuals with diabetes compared to those without diabetes, and the biology of peripheral arterial disease may be different in people with diabetes given the pervasive influence that diabetes has on the atherosclerotic milieu of the peripheral vasculature. Consequently, strategies to prevent and treat peripheral arterial disease among adults with diabetes, in particular, are needed. One potential strategy is the promotion of physical activity, as physical activity is inversely associated with key parameters (e.g., inflammation) that influence peripheral arterial disease among the general population [5], as well as those with diabetes [6].

Indeed, research using self-report physical activity methodology has demonstrated an association between physical activity and peripheral arterial disease [7,8], with these studies generally showing that those with peripheral arterial disease engage in less self-reported physical activity than those without the disease. This association may be bidirectional. It is conceivable that physical activity may help to prevent and treat peripheral arterial disease through, for example, modulation of inflammation and endothelial function. Although some individuals with peripheral arterial disease are asymptomatic, adults with this condition may be less active as a result of disease-induced functional limitations and pain resulting from claudication [7,9]. Encouragingly, however, physical activity has been shown to improve physical functioning [10] and increase distance to onset of claudication pain [11], with the latter likely as a result of physical activity-induced increased blood flow to the periphery and enhanced economy of movement.

Although an association between physical activity and peripheral arterial disease has been established, shortcomings among this area of inquiry include the exclusive utilization of self-report physical activity methodology. This is particularly problematic as self-reported physical activity is prone to considerable measurement error, likely as a result of biases associated with recall, social desirability, item-interpretation, and questionnaires not always being age and culturally tailored [12]. Further, in the general population, validation studies examining the association between self-report physical activity and some gold-standard (e.g., accelerometry) typically show a poor correlation in the range of 0.3-0.5 [13]. We are unaware of any study specifically examining the agreement between self-reported and objectively-measured physical activity among those with peripheral arterial disease. However, research among adults with other chronic diseases that influence peripheral arterial disease, such as obesity [14-16], demonstrates poor agreement between self-reported and objectively-measured physical activity [17]. This increased measurement error associated with self-report physical activity methodology creates serious challenges in delineating physical activity-disease associations and use of self-report methodology may provide inaccurate estimates of physical activity among those with chronic disease. Given the poor agreement between self-reported and objectively-measured physical activity among those with chronic disease, studies focusing on those with chronic disease (including peripheral arterial disease) are encouraged to use an objective measure of physical activity.

Another area in this line of inquiry needing further attention is the population in which the association between physical activity and peripheral arterial disease has been examined. Despite the greater prevalence of peripheral arterial disease among adults with diabetes, coupled with diabetes-induced exacerbations of this condition [1], we are aware of no studies examining the association between objectively-measured physical activity and peripheral arterial disease among diabetics. Similar to those without diabetes, it is expected that diabetics with peripheral arterial disease would engage in less objectively-measured physical activity than their diabetic counterparts; however, we are aware of no available study to support or refute this assertion. Thus, previous studies on this topic may have limited generalizability to populations (e.g., diabetics) at greatest risk for developing this disease.

To address these major gaps in the literature, the purpose of this study was to examine the association between accelerometer-assessed physical activity and peripheral arterial disease among a nationally representative sample of adults with evidence of diabetes. Accelerometry overcomes limitations of self-report methodology by providing an objective measure of the frequency, intensity, and duration of physical activity.

## Method

### Study design

Data from the 2003–2004 National Health and Nutrition Examination Survey (NHANES) were used. NHANES is an ongoing survey conducted by the National Center for Health Statistics. NHANES evaluates a representative sample of non-institutionalized U.S. civilians, selected by a complex, multistage probability design. Briefly, participants are interviewed in their home and then subsequently examined in a mobile examination center. NHANES data is publically available data, with the authors using NHANES data for secondary analyses. All procedures for data collection were approved by the National Center for Health Statistics ethics review board, and all participants provided written informed consent prior to data collection.

### Selection of participants

Participants of the 2003–2004 NHANES who were chosen to be included in this study were considered to have evidence of diabetes because they self-reported a previous diagnosis of the disease (excluding gestational diabetes mellitus), were taking insulin or other diabetic medications to lower blood glucose, had a glycohemoglobin (HgbA1C) of 6.5% or greater, or had a one-time fasting glucose level of 126 mg/dL or higher.

In the 2003–2004 NHANES cycles, 375 participants had diabetes and ankle brachial index (ABI) data, which was used to determine peripheral arterial disease status. Among these, 254 had complete data on the study variables and constituted the analytic sample. Analyzed participants with evidence of diabetes had a lower body mass index than those with evidence with diabetes that were excluded due to missing accelerometer or covariate data (30.4 vs. 33.1 kg/m2, p = 0.05), but did not differ in other variables (p > 0.05 for all).

### Determination of peripheral arterial disease

Peripheral arterial disease was assessed by examination of the ABI. Detailed procedures of the ABI examination can be found elsewhere [18]. Briefly, participants 40 and older were initially eligible for the ABI examination. Participants were excluded if they had a bilateral amputation or weighed more than 400 pounds (due to equipment limitations). While participants rested in supine position, two systolic blood pressure measurements were made in the right arm (brachial artery) and both ankles (posterior tibial arteries). The right ABI was calculated by dividing the highest systolic blood pressure in the right ankle by the highest blood pressure in the arm; similarly, the left ABI was calculated by dividing the highest systolic blood pressure in the left ankle by the highest blood pressure in the arm [1]. The lower of the ABI readings were used in the present analysis [19]. ABI as an indicator of peripheral arterial disease has been validated against gold-standard angiographically that has a sensitivity and specificity, respectively, of 95% and nearly 100% [20].

There appears to be a U-shaped relationship between ABI and cardiovascular disease morbidity and mortality [21]. For adults with diabetes, an ABI < 1 results in an elevated risk for cardiovascular morbidity and mortality (i.e., greater arterial occlusion); between 1 and 1.4 is considered normal, suggesting no evidence of peripheral arterial disease; and above 1.4 (suggesting poorly compressible vessels) is an independent risk factor for cardiovascular disease morbidity and mortality [21,22]. Only 3 participants had an ABI > 1.4; therefore, the primary analyses combined these 3 participants with the group that had an ABI < 1. As a result, for the primary analyses, participants were classified into two groups: normal ABI (1–1.4) and abnormal ABI (<1 or > 1.4) [9].

### Assessment of physical activity

2003–2004 NHANES participants were asked to wear an ActiGraph 7164 accelerometer during all activities, except water-based activities and while sleeping. Estimates for time spent in moderate-to-vigorous physical activity (MVPA) were summarized in 1-minute time intervals. Minutes with activity counts per minute ≥ 2020 were classified as MVPA [23]. Only those participants with at least 4 days of 10 or more hours/day of accelerometer wear time were included in the analyses in order to ensure that data adequately captured habitual physical activity patterns [23]. To monitor the amount of time the device was worn, nonwear was defined by a period of a minimum of 60 consecutive minutes of zero activity counts, with the allowance of 1–2 minutes of activity counts between 0 and 100 [23].

### Measurement of covariates

Various covariates were included based on previous research demonstrating a link between these variables and physical activity/ABI. These covariates included age, gender, race-ethnicity, body mass index (BMI), comorbidity index, smoking (cotinine), HgbA1C, C-reactive protein, homocysteine, glomerular filtration rate (GFR), microalbuminuria, peripheral neuropathy, physical functioning, and medication use.

Information about age, gender, and race-ethnicity were obtained from a questionnaire. BMI was calculated from measured weight and height (weight in kilograms divided by the square of height in meters). A comorbidity index variable was created to classify number of comorbidities each participant had [24]. Participants were classified as having 0 or 1+ comorbidities based on self-report of the following chronic diseases/events: arthritis, hypertension, coronary heart disease, heart attack, congestive heart failure, stroke, cancer, emphysema, asthma, and chronic bronchitis. Blood samples were taken in the mobile examination center to assess for a variety of biological markers. High sensitivity C-reactive protein concentration was quantified using latex-enhanced nephelometry. Serum cotinine was measured as a marker of active smoking status or environmental exposure to tobacco (i.e., passive smoking). Serum cotinine was measured by an isotope dilution-high performance liquid chromatography/atmospheric pressure chemical ionization tandem mass spectrometry. HgbA1C was measured using the Primus instrument, which is a fully automated glycohemoglobin analyzer using high performance liquid chromatography. Homocysteine, a marker of endothelial function, was measured using the fluorescence polarization immunoassay. Glomerular filtration rate (GFR), an assessment of kidney function, was assessed from the Chronic Kidney Disease Epidemiology equation based on specified race, sex, and serum creatinine level [25]. Microalbuminuria was calculated as urinary albumin concentration (mg/dL) divided by urinary creatinine concentration (g/L), with the following gender-specific cut-points used to denote microalbuminuria: ≥ 21 mg/g for males and ≥ 24 mg/g for females [26].

Similar to other studies [3], participants were defined as having peripheral neuropathy if the examination (using a standard monofilament [5.07 Semmes-Weinstein nylon monofilament]) determined at least 1 insensate area in either foot, which is predictive of ulcers and amputations and has demonstrated high sensitivity and specificity [27]. Participants were considered to have a functional disability if they reported special assistance for walking (e.g., cane), had limitations that prevented them from working, or reported having any difficulty in the five functional disability categories reported elsewhere [28]. Lastly, a binary variable was created with participants classified as taking medications if they self-reported taking insulin, diabetic pills, blood pressure-lowering medication, cholesterol-lowering medication, or anticoagulants.

### Data analysis

All statistical analyses were performed using Stata (version 12.0, College Station, TX) and accounted for the complex survey design by using appropriate sampling weights, primary sampling units, and clustering variables. In an effort to maintain nationally representative estimates, the sample weights for those with 4 or more days of valid accelerometry data were ratio-adjusted to maintain the age, sex, and race-ethnicity distribution of the full sample.

Means and standard errors were calculated for continuous variables and proportions were calculated for categorical variables. To examine the association between accelerometer-assessed MVPA and ABI, negative binomial regression models was employed, as the outcome variable (MVPA time) was expressed in integral minutes and was positively skewed. Rate ratios (RR) from the negative binomial regression reflect the rate of events for each variable in the model while holding the other variables in the model constant [29].

Two negative binomial regression models were computed. The first model was a minimally adjusted model including the following covariates: age, BMI, race-ethnicity, gender, and comorbidity index. Following this, a fully adjusted model was computed, including the following covariates that have previously been shown to correlate with physical activity and/or peripheral arterial disease: age, BMI, race-ethnicity, gender, comorbidity index, cotinine, HgbA1C, C-reactive protein, homocysteine, glomerular filtration rate, microalbuminuria, peripheral neuropathy, physical functioning, and medication use.

With regard to the fully adjusted model, there was no evidence of multicollinearity. Evidence of multicollinearity is likely to exist if there is correlation > 0.8 between two covariates; if the mean variance inflation factor is > 6 or if the highest individual variance inflation factor is > 10; or if the tolerance statistic is < 0.1. For the fully adjusted model, the highest correlation between two covariates was 0.55; the mean variance inflation factor was 1.3; the highest individual variance inflation factor was 1.9; and all individual tolerance statistics were > 0.5. Statistical significance was established as an alpha value < 0.05.

## Results

**Figure 1:**
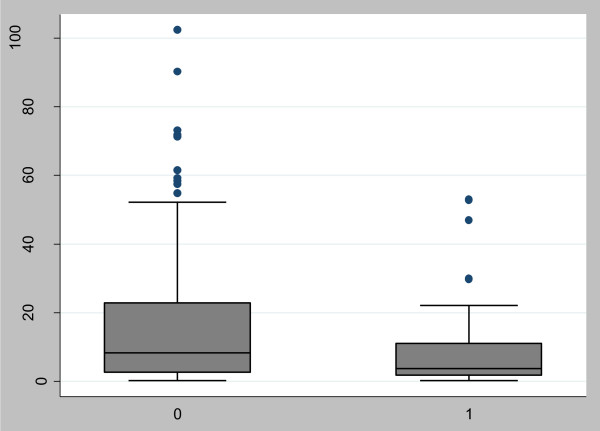
Box-and-whisker plot showing average moderate-to-vigorous physical activity (MVPA) estimates/day (shown on ordinate) across arterial brachial index groups (shown on abscissa; 1 = abnormal arterial brachial index; 0 = normal arterial brachial index).

Participants with normal ABI engaged in 14.3 (SE = 1.3) min/day of MVPA, whereas participants with abnormal ABI engaged in 11.5 (SE = 2.9) min/day of MVPA. The distribution of physical activity across ABI status is shown in Figure 1.

Table 1 reports summary statistics for the study variables among those with normal and abnormal ABI. Participants with abnormal ABI, compared to those with normal ABI, had higher levels of homocysteine and were more likely to have at least one comorbidity.

**Table 1:** Weighted characteristics of analyzed participants among those with normal and abnormal ankle brachial index, NHANES 2003-2004

	**Mean/proportion (95% CI)**		
**Variable**	**Normal ABI (1–1.4) (n = 76)**	**Abnormal ABI (<1 or >1.4) (n = 178)**	***P***
Age (yrs)	60.2 (58.5-61.9)	60.7 (57.2-64.1)	0.74
% Male	57.8 (48.9-66.6)	46.2 (28.8-63.6)	0.22
Body mass index (kg/m^2^)	30.3 (29.4-31.2)	30.6 (27.1-34.1)	0.85
Race-Ethnicity			0.73
% Non-hispanic white	69.1 (56.0-82.1)	70.6 (57.3-83.9)	
% Other	30.8 (17.8-43.9)	29.3 (16.0-42.6)	
Cotinine (ng/mL)	43.4 (19.2-67.7)	68.7 (20.4-117.0)	0.29
HgbA1C (%)	7.1 (6.8-7.4)	7.3 (6.6-7.9)	0.64
C-reactive protein (mg/dL)	0.44 (0.36-0.51)	1.08 (0.33-1.82)	0.09
Homocysteine (umol/L)	9.4 (9.1-9.8)	12.1 (9.8-14.5)	0.04
% Microalbuminuria	30.4 (22.8-38.1)	33.8 (22.7-45.0)	0.63
Comorbidity index			0.0003
% 0 Comorbidities	22.9 (13.3-32.6)	2.2 (0.0-5.5)	
% 1+ Comorbidities	77.0 (67.3-86.6)	97.7 (94.4-100.0)	
% With Peripheral neuropathy	26.0 (15.8-36.3)	15.3 (2.0-28.5)	0.16
% On Medications to treat diabetes, cholesterol, or hypertension	79.4 (73.5-85.2)	86.8 (74.3-99.4)	0.36

An adjusted Wald test was used to examine differences for continuous variables, and a design-based likelihood ratio test was used for categorical variables.

Table 2 shows the results of the negative binomial regression, specifically reporting the independent effects of ABI on MVPA. Both the minimally adjusted and fully adjusted models were significant. In the fully adjusted model, participants with abnormal ABI engaged in 23% less MVPA time (RR = 0.77, 95% CI: 0.62-0.96) than those with a normal ABI. Additionally, and although not shown in tabular format, our findings also demonstrate an association between C-reactive protein and ABI. After all adjustments noted in Table 2, there was an inverse association between ABI and log-transformed C-reactive protein (coefficient = −1.68; 95% CI: −2.55 to −0.81; p = 0.001).

**Table 2:** Negative binomial regression results examining the association between physical activity and ankle brachial index, NHANES 2003-2004

	**Rate ratio (95% CI)**	
**Variables**	**Model 1 minimally adjusted**	**Model 2 fully adjusted**
Ankle brachial index		
Normal (1–1.4)	Referent	Referent
Abnormal (<1 or >1.4)	**0.69 (0.53-0.89)**	**0.77 (0.62-0.96)**
*Covariates*		
Age, 1 year older	**0.95 (0.94-0.96)**	**0.95 (0.93-0.98)**
Gender		
Male	Referent	Referent
Female	**0.61 (0.44-0.85)**	**0.66 (0.45-0.96)**
Body mass index, 1 kg/m^2^ higher	**0.95 (0.93-0.97)**	**0.96 (0.95-0.97)**
Race-ethnicity		
Non-hispanic white	Referent	Referent
Other	1.33 (0.96-1.83)	**1.41 (1.08-1.85)**
Comorbidity index		
0 Comorbidities	Referent	Referent
1+ Comorbidities	1.13 (0.77-1.65)	1.27 (0.90-1.79)
Cotinine, 1 ng/mL higher	N/A	1.00 (0.99-1.01)
HgbA1C, 1% higher	N/A	0.99 (0.90-1.09)
C-reactive protein, 1 mg/dL higher	N/A	**0.88 (0.84-0.93)**
Glomerular filtration rate, 1 unit higher	N/A	0.99 (0.98-1.01)
Homocysteine, 1 umol/L higher	N/A	0.98 (0.94-1.03)
Microalbuminuria		
No	Referent	Referent
Yes	N/A	0.97 (0.77-1.23)
Physical functioning		
No functioning limitations	Referent	Referent
Some functioning limitations	N/A	**0.69 (0.51-0.94)**
Peripheral neuropathy		
No peripheral neuropathy	Referent	Referent
Having peripheral neuropathy	N/A	1.13 (0.60-2.11)
Medications		
Yes	N/A	0.92 (0.60-1.43)
No	Referent	Referent

Model 1 (minimally adjusted) controlled for age, gender, body mass index, race-ethnicity, and comorbidity index.

Mode l 2 (fully adjusted) controlled for age, gender, body mass index, race-ethnicity, comorbidity index, cotinine, HgbA1C, C-reactive protein, glomerular filtration rate, homocysteine, microalbuminuria, physical functioning, peripheral neuropathy, and medication use.

N/A = Not applicable; not entered into model.

Bold indicates statistical significance (p < 0.05).

Given that ABI values > 1.4 are considered an independent risk factor for cardiovascular morbidity and mortality, and although acknowledging the limited sample size, secondary analyses were computed that separated these individuals from those with a low ABI (i.e., < 1). Compared to those with normal (14 min/day) and low (11 min/day) ABI values, the 3 participants with a high ABI (i.e., > 1.4) engaged in the fewest minutes of MVPA (4.8 min/day). However, when these 3 participants were excluded from the fully adjusted negative binomial regression model, the results were unchanged (data not shown).

Lastly, additional analyses were computed to see if duration of diabetes influenced the relationship between MVPA and ABI. Only participants who self-reported having diabetes (n = 188) were asked how long they have had diabetes. Among these 188 participants, and after adjustments along with duration of diabetes, the results were similar (RR = 0.66; 95% CI: 0.44-0.99).

## Discussion

The present findings are the first to demonstrate an association between accelerometer-assessed physical activity and ABI among a nationally representative sample of U.S. adults with evidence of diabetes. These data showed that, after adjustments, adults with diabetes and an abnormal ABI engaged in 23% less MVPA time than their counterparts with a normal ABI. Consequently, adult diabetics with peripheral arterial disease may be at an increased risk of various chronic diseases associated with physical inactivity.

A limitation of the present study is the cross-sectional study design, which precludes any ability to render causation. Diabetics with peripheral arterial disease were clearly less active than their counterparts, which suggest that this condition may limit exercise tolerance, possibly a result of pain duration ambulation. However, it is also plausible to suggest that more active diabetics were less likely to develop peripheral arterial disease due to the benefits of physical activity. The present study cannot determine the direction of association between physical activity and peripheral arterial disease, but to spawn the development of future work on this topic, the narrative that follows will discuss the possibility of physical activity reducing the risk of peripheral arterial disease incidence.

Diabetes may increase an individual’s risk for developing peripheral arterial disease through a variety of underlying mechanisms, all of which are favorably associated with physical activity behavior. Most patients with diabetes demonstrate abnormalities in endothelial function and vascular regulation, which increases the local inflammatory state of the vascular wall [1]. Previous studies have demonstrated a favorable association between physical activity and endothelial function [30], with this association likely occurring through physical activity-induced changes in methionine catabolism, vitamin B availability, inhibition of platelet aggregation and coagulation, and improvements in blood rheology [31-33].

C-reactive protein, a biomarker of inflammation, has been established as a risk marker and may exacerbate peripheral arterial disease [34], and C-reactive protein levels tend to be elevated among those with diabetes [35]. Previous studies have demonstrated an inverse association between physical activity and C-reactive protein [36], with the underlying mechanism likely occurring through changes in adiposity, which would help decrease cytokine production (e.g., IL-1, TNF-alpha), along with improvements insulin resistance and endothelial function [5]. Indeed, in the present study, accelerometer-assessed physical activity was inversely associated with C-reactive protein (Table 2). Our findings also demonstrate an association between C-reactive protein and ABI (noted in the results section), suggesting that physical activity-induced changes in C-reactive protein may, in part, help to explain the association between physical activity and ABI.

In addition to their being biological plausibility to explain the association between physical activity and peripheral arterial disease, clinical exercise trials demonstrate that exercise therapy among patients with peripheral arterial disease may improve walking tolerance, walking performance, walking economy, modulate inflammatory/hemostatic markers, enhance vasoresponsiveness, and induce lower-extremity adaptations (e.g., mitochondrial synthesis, angiogenesis) that may increase oxygen delivery and enhance metabolic responses [37,38]. Not only may physical activity help to reduce the risk of developing peripheral arterial disease, but these findings underscore the importance of promoting physical activity to those with this condition to help attenuate the progression of the disease.

In conclusion, to our knowledge, no study has examined the association between objectively-measured physical activity and peripheral arterial disease in a national sample of adults with diabetes. Our findings demonstrate that adult diabetics with peripheral arterial disease engage in less physical activity than their counterparts. Strengths of this study include this novel examination among this understudied population, using an objective-measure of physical activity, and employing a national sample of adults with diabetes. A limitation includes the cross-sectional design; therefore, future prospective and/or experimental work among diabetics with peripheral arterial disease is needed, while ensuring the utilization of an objective-measure of physical activity. Clinicians are encouraged to promote safe forms of physical activity among diabetics with peripheral arterial disease as these individuals may be at an increased risk of various chronic diseases associated with physical inactivity. Using prospective and/or experimental designs, it would also be informative to see if regular physical activity may help to prevent peripheral arterial disease and attenuate the progression of the disease.

## Competing interests

All authors declare that they have no competing interest.

## Authors’ contributions

Both authors made substantive contributions to the conception of the study, interpretation of the data, were involved in drafting or revising the manuscript, have given final approval of the version to be published, and agree to be accountable for all aspects of the work.
